# ATRA-mediated RAR-α activation attenuates acrylamide-induced testicular toxicity

**DOI:** 10.1038/s41598-026-50168-z

**Published:** 2026-05-07

**Authors:** Hamada Ahmed Mokhlis, Mohammed Helmy Rashed, Ibrahim Ghalib Saleh, Mahmoud Gomaa Eldeib, Ahmed A. El-Husseiny, Emad Gamil Khidr, Maher H. Gomaa, Hesham S. Gad, Mohamed R. Elnagar, Mahmoud Mohamed Mokhtar, Ahmed Aglan

**Affiliations:** 1 Department of Pharmacology and Toxicology, Faculty of Pharmacy, Al- Azhar University, 11884 Cairo, Egypt; 2 Department of Pharmacy Practice, Faculty of Pharmacy, Sinai University- Kantara Branch, Ismailia City, 41636 Egypt; 3 Department of Clinical Pharmacy, Faculty of Pharmacy, Al-Azhar University, 11884 Cairo, Egypt; 4 Biochemistry and Molecular Biology Department, Faculty of Pharmacy, Al- Azhar University, Nasr City, Cairo, 11231 Egypt; 5 Department of Biochemistry, Faculty of Pharmacy, Sinai University- Kantara Branch, Ismailia City, 41636 Egypt; 6 Department of Biochemistry, Faculty of Pharmacy, Egyptian Russian University, Badr City, Cairo, 11829 Egypt; 7 Department of Pharmacology, College of Pharmacy, The Islamic University, Najaf, 54001 Iraq

**Keywords:** All-Trans Retinoic Acid (ATRA), Acrylamide, Testicular Toxicity and Retinoic Acid receptor Alpha (RAR- α), Biochemistry, Cell biology, Medical research, Physiology, Zoology

## Abstract

Acrylamide (ACR) is an environmental reproductive toxicant with unclear testicular toxicity mechanisms. Retinoids are a group of vitamin A-related compounds that function by activating retinoid receptors. We aimed in this study to further explore All-trans retinoic acid’s (ATRA) protective response against an acrylamide-induced testicular insult model and the underlying possible mechanisms. Fifty male rats were allocated into control, DMSO, ACR (40 mg/kg bwt, i.p. daily for 14 days), ATRA (7.5 mg/kg bwt, i.p. daily), and ACR + ATRA groups. Body and testes weights, sperm parameters, testosterone level, and lactate dehydrogenase-X (LDH-X) activity were measured. In addition, testicular levels of glutathione (GSH), superoxide dismutase (SOD), catalase (CAT), malondialdehyde (MDA), tumor necrosis factor-alpha (TNF-α), interleukin-1β (IL-1β), interleukin-6 (IL-6), caspase-3, Bax, and Bcl-2 were determined. Also, tissues were examined for histopathologic changes and immune expression of retinoic acid receptor-alpha (RAR-α). ACR exposure led to reduced body and testicular weights, impaired sperm parameters, and suppressed reproductive hormones (testosterone, FSH, LH). Testicular LDH-X activity decreased, along with reduced testicular RAR-α expression. Oxidative stress resulted in GSH depletion, reduced CAT and SOD activities, and increased MDA. ACR also triggered inflammation and apoptosis, with elevated TNF-α, IL-1β, IL-6, caspase-3, Bax. In contrast, ATRA improved sperm parameters and levels of hormones, restored RAR-α expression, mitigated oxidative stress, and decreased inflammation and apoptosis markers. Morphometric and histopathologic studies supported these biochemical observations. Overall, RAR-α agonist (ATRA) is linked to attenuation against ACR-induced testicular damage, along with a reduction in oxidative stress, inflammation, and cell death. These findings suggest that retinoid signaling might serve as a possible therapeutic target for reproductive toxicities induced by ACR, necessitating further mechanistic exploration.

**Supplementary Information:**

The online version contains supplementary material available at 10.1038/s41598-026-50168-z.

## Introduction

Acrylamide is an organic substance with substantial applications in industrial, environmental, and medical fields^1^. It is primarily utilized to synthesize polyacrylamide, a polymer with applications in water treatment, paper manufacturing, improved oil recovery, and mining^2^^3^. It is also formed when foods rich in carbohydrates are exposed to high temperatures during culinary procedures including frying, baking, and roasting. Notably, it is an outcome of the chemical reaction between reducing sugars and amino acids known as the Maillard reaction^4^.

Exposure to acrylamide can happen through environmental contamination, occupational contact, and food consumption^5^. In both humans and animals, long-term exposure has been associated with peripheral neuropathy, cognitive impairments, motor dysfunction, detrimental outcomes on male reproduction, and increased cancer risk in various organs^6–9^^10^. After consumption, the cytochrome P450 enzymes in the liver predominantly metabolize acrylamide, producing the genotoxic metabolite glycidamide. The mutagenic and carcinogenic potential of glycidamide is attributed to the formation of DNA adducts^11^. According to several in vivo studies, rats exposed to acrylamide over an extended period have been shown to develop testicular toxicity^12^, liver and kidney toxicity^13^, and neurotoxicity^14^. Acrylamide’s toxic consequences are mainly mediated by the induction of oxidative stress, which can hamper immune function and promote inflammation^15^. In testicular tissue specifically, acrylamide causes significant damage by inducing oxidative stress, depleting protective molecules like GSH, SOD, and CAT, and increasing harmful MDA, leading to mitochondrial dysfunction and germ cell death via the Bax/Bcl-2/caspase-3 pathway. It also activates inflammation via the NF-κB pathway, damaging the blood-testis barrier and Sertoli and Leydig cells^15^^16^^17^.,.

Carotenoids and vitamin A have powerful antioxidant characteristics. Retinal, retinol, and retinoic acid (RA) are the body’s forms of vitamin A^18^. Through its chain-breaking action, retinoic acid suppresses the generation of free radicals and hinders them without interaction with biologically relevant targets^19^. Notably, all-trans retinoic acid (ATRA) represses lipid peroxidation in the cellular plasma membranes. Besides, it diminishes inflammation and cellular proliferation, and exerts anticancer activity^20^^21^. There are two distinct groups of retinoid receptors documented to have evolved: the retinoid X receptors (RXRs), which exclusively recognize 9-cis RA, and the RA receptors (RARs), which detect both 9-cis RA and ATRA stereoisomers. Additionally, there are three distinct forms of receptors in each group: α, β, and γ^22^.

Although acrylamide is known to cause testicular damage, including decreased sperm count, hormonal imbalance, oxidative stress, and apoptosis, the precise signaling pathways and molecular starting events involved are still unclear. The major research gap is the absence of a clear, mechanistic connection between acrylamide exposure and the dysregulation of particular nuclear receptors, which are master regulators of steroidogenesis, antioxidant defense, and testicular function. As a result, our preliminary research showed that retinoic acid receptor (RAR) expression is downregulated in testicular tissues following acrylamide exposure during screening trials for certain nuclear receptors. Therefore, we suppose that RAR agonist (ATRA) may have a role in modulating acrylamide actions. To the best of our knowledge, the impact of ATRA on acrylamide-mediated testicular toxicity has not been assessed yet. Thus, this in vivo study was conducted to evaluate the possible mitigating effect of ATRA towards acrylamide-mediated testicular toxicity and elucidate some possible fundamental mechanisms that could be involved.

## Materials and methods

### Materials

Acrylamide and All Trans-Retinoic acid (ATRA) were purchased from Sigma-Aldrich (St Louis, MO, USA).

### Animals

Fifty Sprague-Dawley rats, aged 14–15 weeks and weighing between 140 and 150 g, were obtained from El-Nile Company in Cairo, Egypt. Rats were kept in well-ventilated enclosures under stable conditions of 22 ± 2 °C room temperature, 50–70% humidity, and a 12-hour light/dark cycle. Animals were provided with a regular diet and access to water as needed, and they had a week to adjust to the new environment prior to the start of the experiment.

### Experimental design

At the start of the experiment, rats were weighed and randomly assigned to five groups (10 rats each): one group received normal saline (control group), other groups received 0.1 ml/kg (DMSO), 40 mg/kg of ACR (ACR group)^16^, 7.5 mg/kg of ATRA^23,24^^25^(ATRA group), and the final group received 7.5 mg/kg of ATRA followed by 40 mg/kg of ACR (ACR with ATRA group). Saline, ACR, and ATRA were given daily via intraperitoneal injection for a continuous period of 14 days. Group allocation was performed by simple randomization using a random number table. Conducting biochemical assays and histopathological assessments were blinded to group assignments throughout the study to minimize observer bias. The sample size of 10 rats per group was determined a priori by power analysis (α = 0.05, power = 0.80). The study is reported in accordance with ARRIVE guidelines (https://arriveguidelines.org). Twenty-four hours post the final dose, the animals were weighed and anesthetized with intraperitoneal ketamine (50 mg/kg) and xylazine (5 mg/kg). After which blood samples were drawn from the retro-orbital sinus and centrifuged to isolate the serum, which was then kept at − 80 °C until needed.

### Euthanasia and tissue collection

All animals were euthanized under deep anesthesia via an overdose of ketamine (80 mg/kg) and xylazine (10 mg/kg). No outdated agents (e.g., chloral hydrate, ether, or chloroform) were used. All experimental protocols were reported in accordance with the ARRIVE guidelines (https://arriveguidelines.org). (PLoS Biol 8(6), e1000412, 2010). The testes along with the caudal epididymis were removed, weighed, and rinsed with ice-cold saline. The right testis from each rat was prepared for histological assessment, while the left testis was homogenized in ice-cold 0.15 M KC1 (10% w/v), subsequently centrifuged at 9,000 g for 10 min at 4 °C, and the supernatant was promptly gathered and utilized for biochemical analysis and enzyme activity measurements. All methods were performed in accordance with the relevant guidelines and regulations.

### Determination of sperm parameters

The epididymis was finely minced with anatomical scissors in 5 mL of physiological saline, placed on a rocker for 10 min, then allowed to sit at room temperature for 2 min. A single drop of sperm suspension was put on a glass slide to examine 200 motile sperm across 4 various fields. The motility of the sperm from the epididymis was assessed with a phase-contrast microscope at a magnification of 400x within 2 to 4 min after their extraction from the epididymis. Motility was defined as any detectable movement of the sperm flagellum within a 30-second observation window, and the motility rate was represented as a percentage of motile sperm relative to the total sperm count. The overall sperm count was assessed using a Neubauer hemocytometer as outlined by *Yokoi et al.*^26^.

### Enzyme linked immunosorbent assay (ELISA) for all biochemical parameters

According to the manufacturer’s guidelines, the standard and serum samples were introduced and adsorbed into the wells of an ELISA plate, which were then covered with microplate sealers and incubated at 37 °C for 30 min. Subsequently, the wash buffer was utilized to rinse the plate five times (30 s each), followed by the addition of 50 µl of horseradish peroxidase (HRP)-conjugated reagent to each well, incubating at 37 °C for 30 min. The plate was then washed five more times, after which 50 µl of TMB substrate solutions A and B were added alternately to each well and incubated at 37 °C for 10 min^27^.

### Determination of serum hormone levels

Serum hormonal levels were measured using the rat testosterone, FSH and LH competitive ELISA kit (LifeSpan Bioscience, Inc, USA) with catalog number. LS-F28083, LS-F6305 and LS-F20636, respectively, following the manufacturer’s guidelines.

### Determination of Lactate dehydrogenase isoenzyme-x (LDH-X)

Lactate dehydrogenase isoenzyme-x (LDH-X) was assessed in the supernatant of tissue homogenate utilizing the Rat LDH-X ELISA Kit (MyBioSource, CA, USA) with catalog No. MBS3808917 follows the guidelines set by the manufacturer.

### Determination of testicular oxidative stress

Lipid peroxidation was assessed by evaluating the MDA levels through the thiobarbituric acid (TBA) test^28^. The MDA concentration was represented as nmol/g of tissue. Reduced glutathione was evaluated using the method of Beutler and Mary^29^with a colorimetric assay kit (Bio-Diagnostic, Egypt), following the manufacturer’s guidelines, and GSH levels were reported as mg/g of tissue. The activities of the antioxidant enzymes CAT and SOD in the testis were assessed using the method of Aebi, H^30^. and^31^ respectively, through a colorimetric assay kit (Bio-Diagnostic, Egypt) following the manufacturer’s guidelines; the activities were reported as U/g of tissue.

### Immunofluorescence staining for determination of retinoic acid receptors (RAR-α)

Procedures for immunofluorescence staining were carried out using Abdel-Bakky et al.‘s methodology^32^. In short, tissue slices that had been paraffinized were dissolved in an oven set to 60 °C for 20 min, and then they were de-paraffinized in 100% xylene for 15 min. The sections were rehydrated by immersing them for five minutes in ethanol concentrations that were graded: 100%, 90%, 70%, 50%, and 30%. Tissue slices were rehydrated, rinsed with distilled water for 15 min, and then incubated for 20 min in a microwave oven using Dako solution (0.01 M sodium citrate buffer, pH 6). Following cooling, tissue slices were fixed with methanol for 10 min, blocked with blocking buffer for 1 h at room temperature, and then rinsed for 15 min with phosphate-buffered saline containing 0.05% TWEEN 20 (PBST) at pH 7.4. The tissue sections were then treated for the entire night at 4 °C in a cool, dark environment with the primary antibodies (mouse polyclonal RAR-α “sc-515796”) at a concentration of 1:500. Following three 5-minute PBST washes of the stained sections, secondary antibodies (Alexa Fluor 488) were used for 30 min to identify the bound antibodies. Following a 30-minute PBST wash, the nuclei were counterstained with 4′,6′-diamidino-2-phenylindole (DAPI) fluorescent stain. Lastly, tissue sections were examined using a Leica DM5000 B fluorescent microscope (Leica, Germany) and mounted using the anti-fade mounting solution Fluoromount^®^ (Sigma Aldrich, St. Louis, MO). Using Image-J/NIH software, the average fluorescence intensity of three to five microscopic fields was determined for every tissue section and adjusted to DAPI intensity.

### Determination of testicular proinflammatory cytokines (TNF-α, IL-1β and IL-6)

Using an ELISA kit specifically designed for rats (Cloud-Clone Corp., Houston, USA) with catalog numbers SEA133Ra, SEA563Ra, and SEA079Ra, the amounts of TNF-α, IL-1β, and IL-6 in testicular tissue homogenate were estimated. The test methods were carried out according to the manufacturer’s instructions.

### Determination of testicular apoptotic markers (Caspase-3, Bax and Bcl-2)

Using an ELISA kit specifically designed for rats (Cloud-Clone Corp., Houston, USA) with catalog numbers SEA626Ra, SEB343Ra, and SEA778Ra, the amounts of Caspase-3, Bax and Bcl-2 in testicular tissue homogenate were estimated. The test methods were carried out according to the manufacturer’s instructions.

### Isolation of total RNA, synthesis of cDNA, and analysis via real-time PCR in testicular tissue

Total RNA extraction from testicular tissue was conducted using QIAzol Lysis Reagent (Qiagen, Cat: 79306, Germany). The manufacturer’s guidelines for isolation were followed meticulously, in sequence. The total RNA concentrations measured were assessed using the NanoDrop device (BIO-TEK INSTRUMENTS EPOCH, USA), and total RNA normalization was conducted based on the results obtained. Subsequently, cDNA synthesis was conducted from the total RNAs utilizing the iScript™ cDNA Synthesis Kit (BIO-RAD, USA), following the guidelines provided by the manufacturer. The resulting cDNAs were employed to measure the mRNA transcript levels of the genes listed in **Supplementary Table 1.** During the RT-PCR phase, the primers for the genes of caspase-3, Bax and Bcl-2 were created by combining the QuantiTect SYBR Green PCR Master Mix (Qiagen, Cat: 204143, Germany), RNase-free water, and the cDNAs. Reactions were performed in triplicate utilizing the ROTOR-GENE Q (Qiagen, Germany). Internal control was established with β-actin, and the fold change from the CT values acquired was calculated using Livak and Schmittgen’s 2 − ΔΔ^CT^ method.

### Histopathological assessment

Following Bancroft and Gamble’s procedure^33^,. testes were preserved in Bouin’s solution, and tissue was processed and embedded in paraffin blocks to provide 5-µm thick sections that could be used to examine histological changes using hematoxylin and eosin stain. Following that, a light microscope (Olympus BX 41, Japan) was used to view and take pictures of the stained sections.

Spermatogenesis was quantitatively assessed using the Johnsen score^34^ by evaluating 30 seminiferous tubules per animal. Each tubule was assigned a score from 1 to 10 as follows: 10 = complete spermatogenesis with many spermatozoa; 9 = many spermatozoa but disorganized epithelium; 8 = only few spermatozoa; 7 = no spermatozoa but many spermatids; 6 = only few spermatids; 5 = no spermatozoa/spermatids but many spermatocytes; 4 = only few spermatocytes; 3 = only spermatogonia; 2 = only Sertoli cells; 1 = no cells in tubular lumen. The mean score per animal was calculated and used for statistical comparison. The severity of testicular injury was also graded according to the Cosentino scoring system^35^^36^ based on the degree of germinal epithelial damage:

**Grade 1** - Normal testicular architecture with orderly germ cell arrangement;

**Grade 2** - Less orderly, non-cohesive germ cells and closely packed tubules;

**Grade 3** - Disordered, sloughed germ cells with shrunken pyknotic nuclei and less distinct seminiferous tubule borders;

**Grade 4** - Seminiferous tubules closely packed with coagulative necrosis of germ cells.

###  Statistical analysis

All statistical analysis was performed using GraphPad Prism software, version 6. Data were expressed as mean ± SD, where SD stands for standard deviation (SD). Data normality was confirmed using the Shapiro-Wilk test; Homogeneity of variance was assessed using Brown-Forsythe test. Differences between obtained values were compared by one-way analysis of variance (ANOVA) followed by Tukey’s test as *a post hoc* for multiple comparisons. Differences were considered statistically significant at ^a^ *p* < 0.05 versus control, ^b^ *p* < 0.05 versus DMSO, ^c^ *p* < 0.05 versus ACR, ^d^ *p* < 0.05 versus ATRA + ACR.

## Results

### Effect of ACR and ATRA on body and testis weights

The results in Table 1 illustrate the effects of ACR and ATRA treatments on the body and testes weights.

Administration of ACR for 14 consecutive days significantly decreased the final BW (12.8%), percent of weight gain (53.27%), final absolute testes weight (24.6%) and relative weight of testes (29.11%), compared to normal control group. At the same time, treatment with ATRA for 14 consecutive days did not show any significant effect on weight parameters. However, treatment with ATRA before ACR administration resulted in a significant increase in final BW (8.5%), BW gain (88.45%), final absolute testes weight (32.3%) and the relative testes weight (16.38%) with respect to ACR alone treatment. **(**Table 1**).**

**Table 1:**
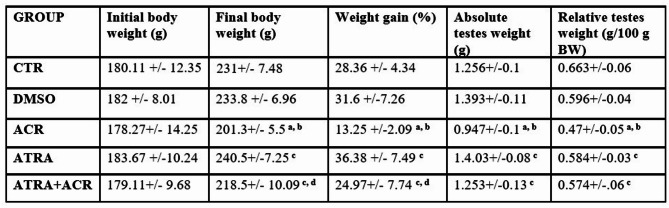
Effect of ATRA and/or ACR body and testes weights. The effects of ACR and/or ATRA on the body and testes weights. Data are mean ± SD; *N* = 10. ^a^ Significant from CTR. ^b^ Significant from DMSO. ^c^ Significant from ACR. ^d^ Significant from ATRA at *P* < 0.05, one-way ANOVA followed by post-hoc Tukey's test. One-way ANOVA revealed significant treatment effects on all weight parameters, including final body weight [F(4, 45) = 10.93, *p* = 0.001], body weight gain [F(4, 45) = 9.29, *p* = 0.001], absolute testis weight [F(4, 45) = 3.535, *p* = 0.014], and relative testis weight [F(4, 45) = 3.144, *p* = 0.023].

### Impact of ACR and ATRA on sperm parameters (count and motility)

**Fig. 1:**
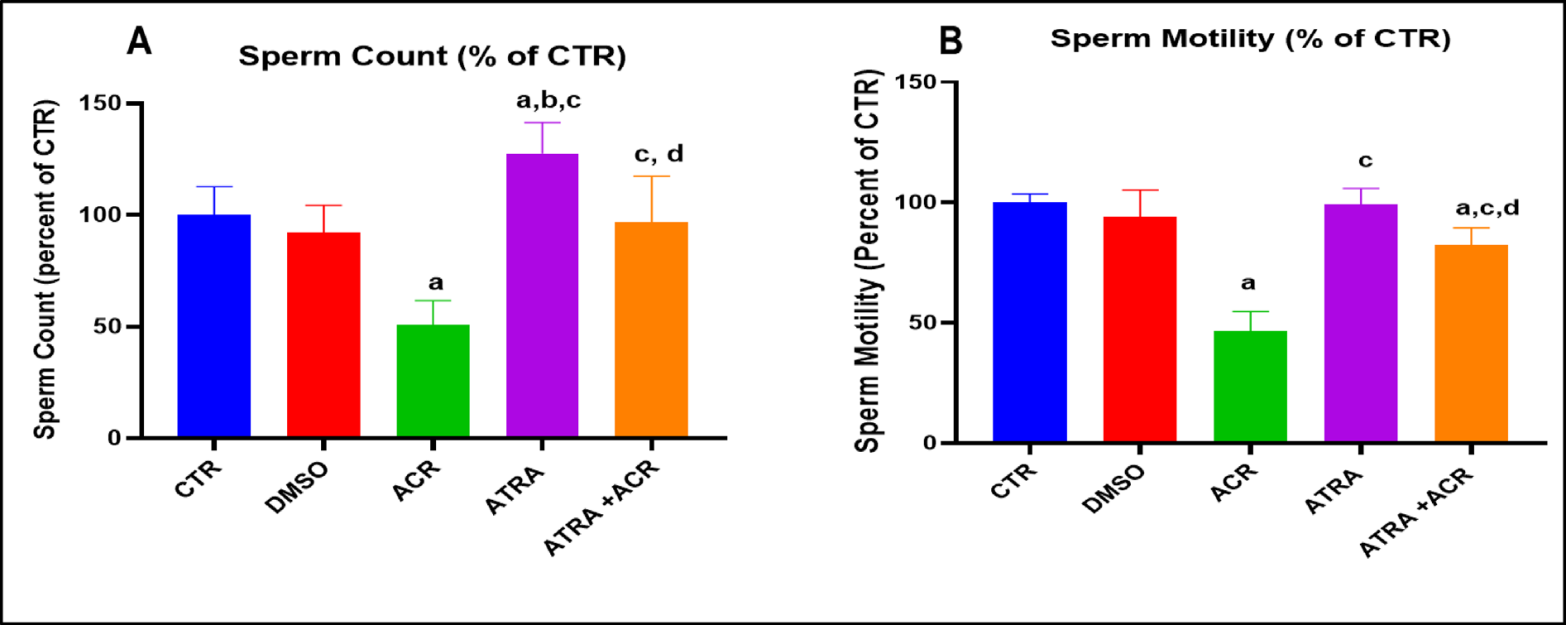
Percentage change of the effect of ATRA and/or ACR on epididymal sperm count (A) and percent of sperm motility (B). The data represent the percentage change of sperm parameters after different treatments. Data are mean ± SD; *N* = 10. ^a^ Significant from CTR. ^b^ Significant from DMSO. ^c^ Significant from ACR. ^d^ Significant from ATRA at *P* < 0.05, one-way ANOVA followed by post-hoc Tukey's test. One-way ANOVA revealed significant treatment effects on sperm count [F(4, 45) = 21.34, *p* < 0.0001] and sperm motility [F(4, 45) = 50.05, *p* < 0.0001].

Data in **Table **2** and** Fig. 1 show the effect of ACR and ATRA treatments on the sperm parameters. Treatment with ACR resulted in a significant decrease in the normal sperm count and motility amounted by about 49% and 44.5%, respectively. Additionally, pre-administration of ATRA before ACR resulted in significant increase in the sperm count and motility by about 89.47% and 77.43%, respectively, with respect to ACR alone treatment.

**Table 2:** Effect of ATRA and /or ACR on epididymal sperm count and percent of sperm motility. The effects of ACR and/or ATRA on sperm parameters. Data are mean ± SD; N = 10.^a^ Significant from CTR. ^b^ Significant from DMSO. ^c^ Significant from ACR. ^d^ Significant from ATRA at P < 0.05, one-way ANOVA followed by post-hoc Tukey's test. One-way ANOVA revealed significant treatment effects on sperm count [F(4, 45) = 28.84, p < 0.0001] and sperm motility [F(4, 45) = 32.05, p < 0.0001].

Group	Sperm count x 10^6 (mean +/- SD)^	Sperm motility (%) (mean +/- SD)
CTR	358.3 +/- 45.46	86.13 +/- 3.02
DMSO	329.2 +/- 44.66	81 +/- 9.5
ACR	182.5 +/- 38.31 ^a^	39.92 +/- 7.24 ^a^
ATRA	456.7 +/- 50.07 ^a,b,c^	85.41 +/- 5.63 ^a^
ATRA+ACR	345.8 +/- 74.73 ^c,d^	70.76+/-6.26 ^a,b,c^

### Effect of ACR and ATRA on serum FSH, LH, testosterone levels and testicular LDH-X activity

**Fig. 2:**
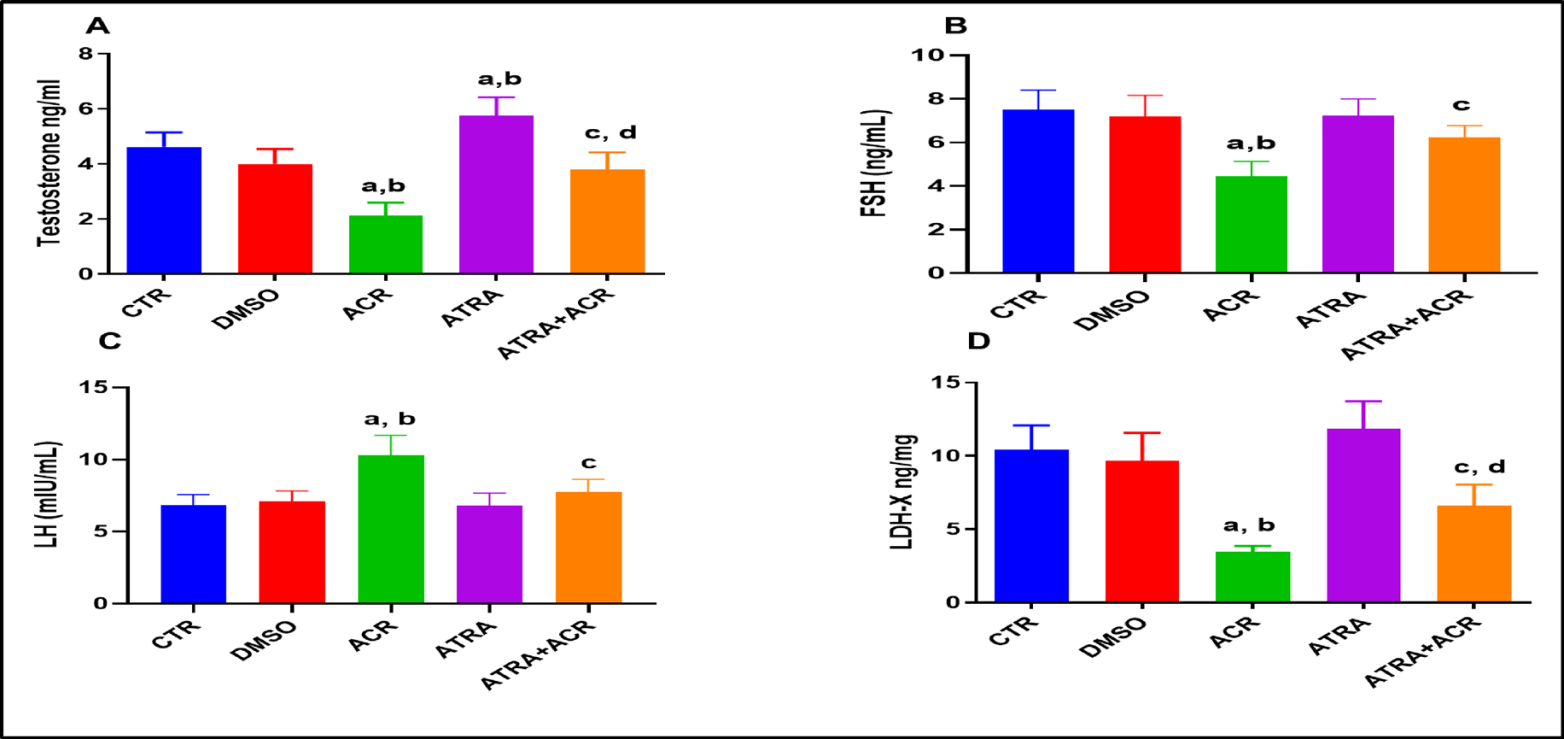
Effects of ATRA and/or ACR administration on the serum testosterone (A), FSH (B), LH (C) and testicular LDH-X (D) in rats. Data are mean ± SD; *N* = 10. ^a^ Significant from CTR. ^b^ Significant from DMSO. ^c^ Significant from ACR. ^d^ Significant from ATRA at *P* < 0.05, one-way ANOVA followed by post-hoc Tukey's test. One-way ANOVA revealed significant treatment effects on serum testosterone [F(4, 45) = 26.48, *p* = 0.0001], serum FSH [F(4, 45) = 15.10, *p* = 0.0001], serum LH [F(4, 45) = 24.76, *p* = 0.0001], and testicular LDH-X activity [F(4, 45) = 28.45, *p* = 0.0001].

Figure 2 illustrates the effects of ACR and ATRA treatments on serum levels of testosterone (A), FSH (B), and LH (C), as well as testicular LDH-X activity (D). ACR treatment significantly reduced serum testosterone levels compared with the control (CTR) group (2.126 ± 0.467 vs. 4.624 ± 0.518 ng/mL), representing a decrease of approximately 54.0% (*p* = 0.0001). In contrast, ATRA treatment alone significantly increased serum testosterone levels compared with the CTR group (5.740 ± 0.683 ng/mL), reflecting an increase of approximately 19.4% (*p* = 0.041). Moreover, ATRA pre-treatment prior to ACR exposure significantly increased serum testosterone levels compared with the ACR group (3.80 ± 0.625 vs. 2.126 ± 0.467 ng/mL), corresponding to an increase of approximately 44.0% (*p* = 0.001).

Similarly, serum FSH levels were significantly reduced in the ACR group compared with the CTR group (4.433 ± 0.686 vs. 7.5 ± 0.903 mIU/mL), representing a decrease of approximately 40.9% (*p* = 0.0001). ATRA co-administration significantly restored FSH levels compared with the ACR group (6.233 ± 0.839 vs. 4.433 ± 0.686 mIU/mL), reflecting an increase of approximately 40.6% (*p* = 0.004).

Conversely, serum LH levels were significantly elevated in the ACR group compared with the CTR group (10.32 ± 1.346 vs. 6.85 ± 1.169 mIU/mL), indicating an increase of approximately 46.4% (*p* = 0.0001). ATRA co-administration significantly attenuated this elevation compared with the ACR group (7.75 ± 0.872 vs. 10.32 ± 1.346 mIU/mL), corresponding to a reduction of approximately 24.9% (*p* = 0.0001), with levels approaching those of the CTR group.

Testicular lactate dehydrogenase isoenzyme X (LDH-X) activity was significantly reduced following ACR treatment compared with the CTR group (3.46 ± 0.401 vs. 10.45 ± 1.63 ng/mg), representing a decrease of approximately 67% (*p* = 0.0001). In contrast, ATRA pre-treatment prior to ACR exposure significantly increased LDH-X activity compared with the ACR group (6.588 ± 1.45 vs. 3.46 ± 0.401 ng/mg), reflecting an increase of approximately 47.5% (*p* = 0.013).

### Effect of ATRA and/or ACR treatment on retinoic acid receptor (RAR-α) protein expression

**Fig. 3:**
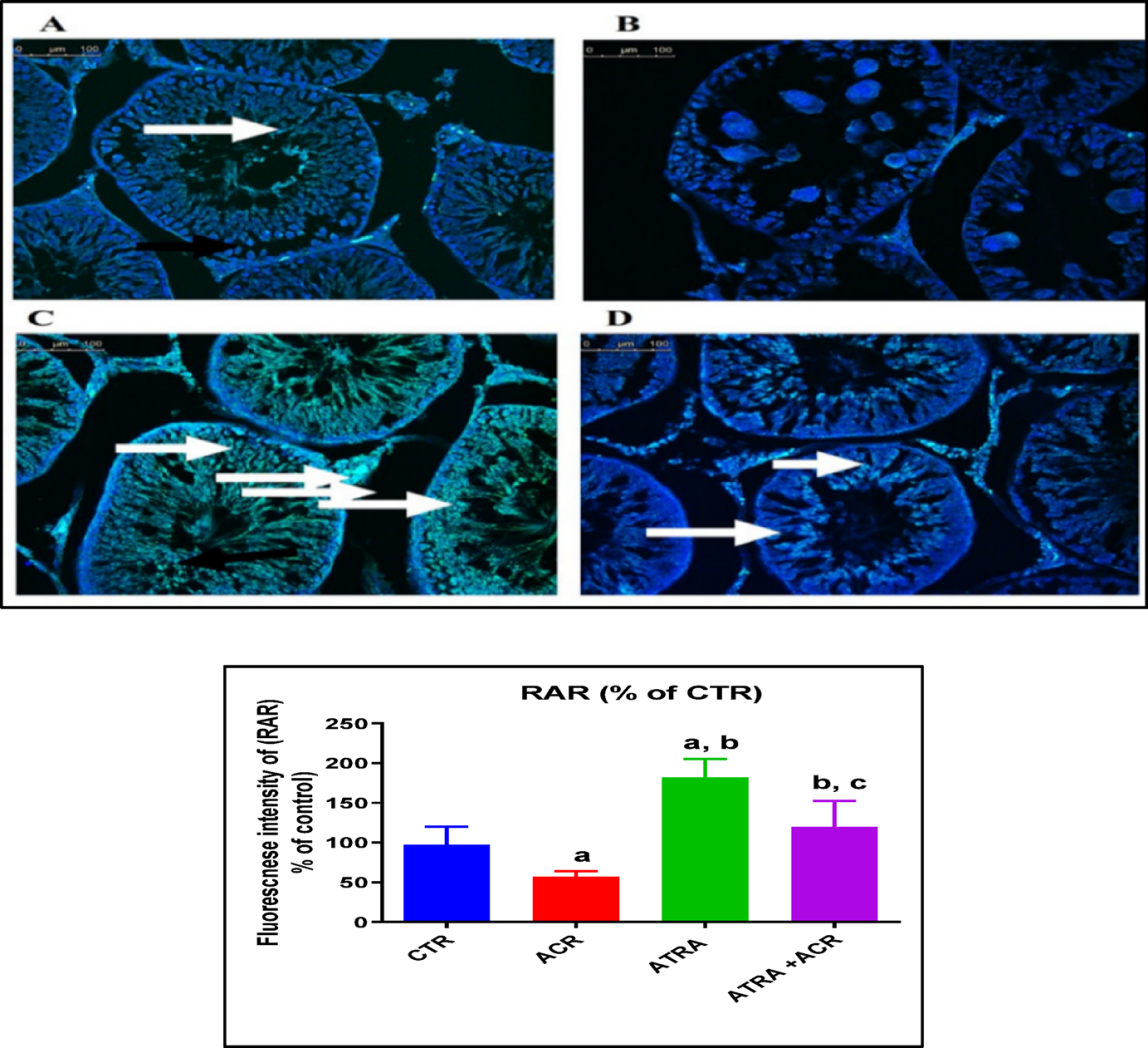
Effects of ATRA and/or ACR on RAR-alpha expression in the testes. The upper panel represents representative fluorescence photomicrographs of testis obtained from control (A), ACR (B), ATRA (C), and ATRA + ACR (D) treated rats, probed for RAR (green fluorescence) (arrows) and DAPI as the counterstain (blue fluorescence). The lower panel shows fluorescence intensity measured from the testes was obtained from five fields of each section (minimally two rats in each group), using ImageJ software. Values are presented as the mean ± SD (*n* = 10). ^a^ Significant from CTR. ^b^ Significant from ACR. ^c^ Significant from ATRA at *P* < 0.05, one-way ANOVA followed by post-hoc Tukey's test. One-way ANOVA identified a significant treatment effect on RAR-α fluorescence intensity [F (3, 36) = 26.8, *p* = 0.001].

The results illustrated in Fig. 3 show that RAR-α protein is constitutively expressed in the seminiferous tubules (A). ACR treatment significantly downregulated RAR-α expression compared with the control (CTR) group (57.26 ± 6.74), representing a decrease of approximately 41.13% (*p* = 0.01). In contrast, ATRA treatment significantly upregulated RAR-α expression compared with the CTR group (182.2 ± 22.94), corresponding to an increase of approximately 87.3% (*p* = 0.0001). Furthermore, co-treatment with ACR and ATRA significantly increased RAR-α expression compared with ACR treatment alone (119.8 ± 32.85), reflecting an increase of approximately 109.2% (*p* = 0.0001).

### Assessment of testicular oxidative stress parameters

**Fig. 4:**
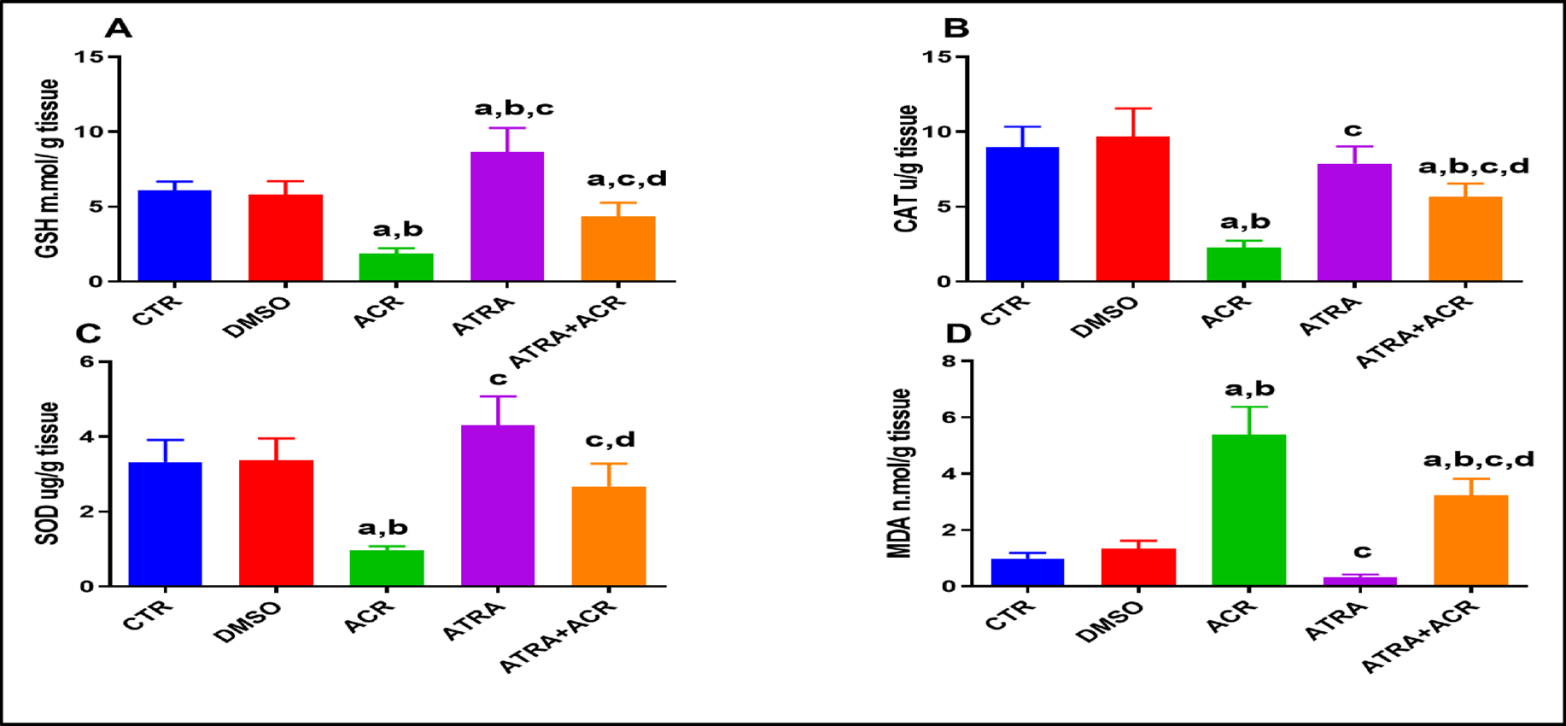
Effects of ATRA and/or ACR on the oxidative stress markers; GSH (A), CAT (B), SOD (C) and MDA (D) levels in testicular tissues of rats treated with acrylamide (ACR). Data are mean ± SD; *N* = 10. ^a^ Significant from CTR. ^b^ Significant from DMSO. ^c^ Significant from ACR. ^d^ Significant from ATRA at *P* < 0.05, one-way ANOVA followed by post-hoc Tukey's test. One-way ANOVA revealed significant treatment effects on GSH [F(4, 45) = 39.69, *p* = 0.0001], MDA [F(4, 45) = 74.58, *p* = 0.0001], SOD activity [F(4, 45) = 27.26, *p* = 0.0001], and CAT activity [F(4, 45) = 35.18, *p* = 0.0001]..

Figure 4 illustrates the effects of ACR and/or ATRA treatments on GSH (A), MDA (B), SOD (C), and CAT (D) levels in testicular tissues. ACR treatment significantly depleted testicular GSH levels compared with the control (CTR) group (1.877 ± 0.354 vs. 6.063 ± 0.605 mmol/g), representing a reduction of approximately 69% (*p* = 0.0001). ATRA pre-treatment significantly restored GSH levels compared with the ACR group (4.348 ± 0.902 mmol/g), corresponding to an increase of approximately 131.6% (*p* = 0.0014).

Conversely, MDA levels were significantly elevated in the ACR group compared with the CTR group (5.394 ± 0.980 vs. 0.984 ± 0.208 nmol/g), reflecting an increase of approximately 448.17% (*p* = 0.0001). ATRA co-treatment significantly reduced MDA levels compared with the ACR group (3.234 ± 0.579 vs. 5.394 ± 0.980 nmol/g), corresponding to a reduction of approximately 40.0% (*p* = 0.0001); however, levels remained significantly higher than those in the CTR group (*p* = 0.0001).

Furthermore, SOD activity was significantly reduced in the ACR group compared with the CTR group (0.963 ± 0.1095 vs. 3.320 ± 0.592 U/g), indicating a decrease of approximately 70.9% (*p* = 0.0001). ATRA pre-treatment significantly restored SOD activity compared with the ACR group (2.665 ± 0.618 vs. 0.963 ± 0.109 U/g), representing an increase of approximately 175.8% (*p* = 0.0003).

Similarly, CAT activity was significantly suppressed in the ACR group compared with the CTR group (2.253 ± 0.480 vs. 8.965 ± 1.383 U/g), corresponding to a reduction of approximately 74.8% (*p* = 0.0001). ATRA pre-treatment significantly enhanced CAT activity compared with the ACR group (5.665 ± 0.884 vs. 2.253 ± 0.480 U/g), reflecting an increase of approximately 151.4% (*p* = 0.0006).

### Effect of ATRA and/or ACR treatment on Pro-inflammatory cytokines

**Fig. 5:**
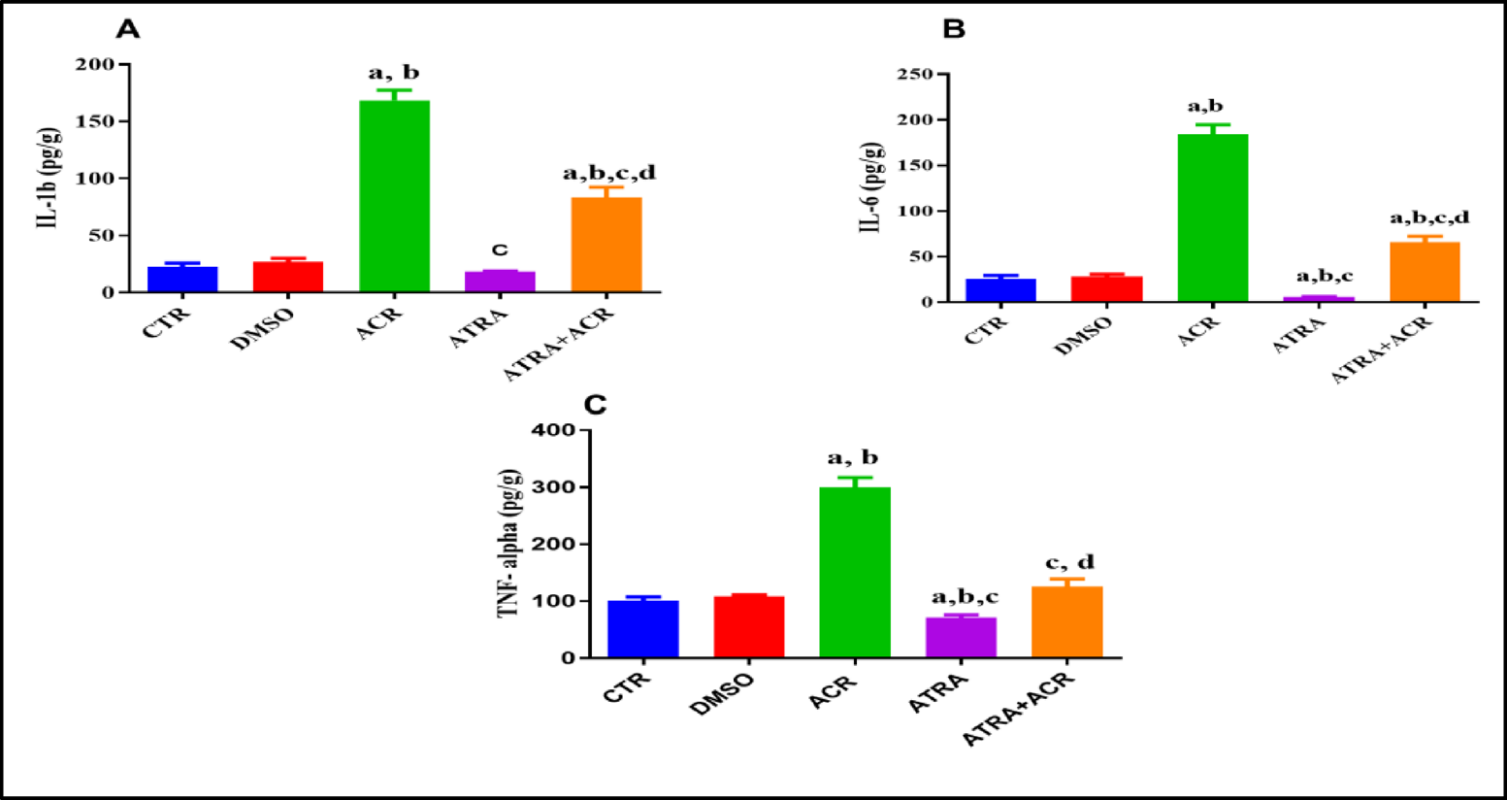
Effects of ATRA and/or ACR on proinflammatory cytokine biomarkers. IL-1β (A), IL-6 (B) and TNF- α (C) levels in testicular tissues of rats treated with acrylamide (ACR). Results expressed as the mean ± SD (*n* = 10). ^a^ Significant from CTR. ^b^ Significant from DMSO. ^c^ Significant from ACR. ^d^ Significant from ATRA at *P* ≤ 0.05, one-way ANOVA followed by post-hoc Tukey's test. One-way ANOVA revealed significant treatment effects on IL-1β [F(4, 45) = 32.00, *p* = 0.0001], IL-6 [F(4, 45) = 39.80, *p* = 0.0001], and TNF-α [F(4, 45) = 23.00, *p* = 0.0001].

As shown in Fig. 5, ACR administration significantly increased testicular levels of IL-1β, IL-6, and TNF-α. Specifically, IL-1β levels were significantly higher in the ACR group compared with the control (CTR) group (168.4 ± 8.968 vs. 22.49 ± 3.316 pg/g), representing an increase of approximately 648.7% (*p* = 0.0001). In contrast, ATRA pre-treatment prior to ACR exposure significantly reduced IL-1β levels compared with the ACR group (83.24 ± 9.167 vs. 168.4 ± 8.968 pg/g), corresponding to a decrease of 50.57% (*p* = 0.0001).

Similarly, IL-6 levels were significantly elevated in the ACR group compared with the CTR group (183.6 ± 11.13 vs. 25.98 ± 3.47 pg/g), showing an increase of approximately 606.7% (*p* = 0.0001). ATRA pre-treatment significantly attenuated this increase, reducing IL-6 levels compared with the ACR group (65.5 ± 6.920 vs. 183.6 ± 11.13 pg/g), corresponding to a reduction of approximately 64.3% (*p* = 0.0001).

In addition, TNF-α levels were significantly higher in the ACR group than in the CTR group (299.9 ± 16.93 vs. 100.9 ± 6.502 pg/g), reflecting an increase of approximately 197.9% (*p* = 0.0001). Conversely, ATRA pre-treatment significantly reduced TNF-α levels compared with the ACR group (125.5 ± 13.16 vs. 299.9 ± 16.93 pg/g), representing a decrease of approximately 58.15% (*p* = 0.0001).

### Effect of ATRA and/or ACR treatment on apoptosis markers

**Fig. 6:**
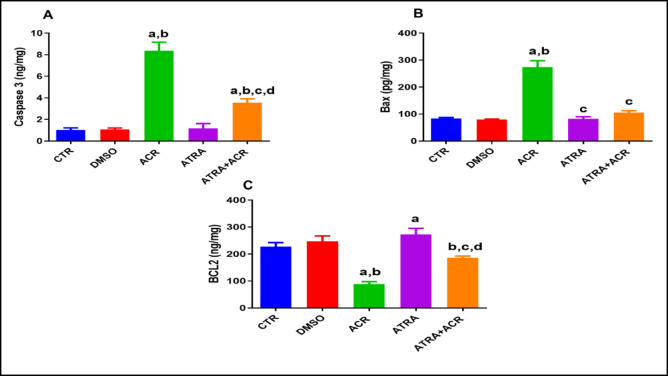
Effects of ATRA and/or ACR on apoptosis biomarkers: Caspase-3 (A), Bax (B), and BCL-2 (C) levels in testicular tissues of rats treated with acrylamide (ACR). Results expressed as the mean ± SD (*n* = 10). ^a^ Significant from CTR. ^b^ Significant from DMSO. ^c^ Significant from ACR. ^d^ Significant from ATRA at *P* < 0.05, one-way ANOVA followed by post-hoc Tukey's test. One-way ANOVA revealed significant treatment effects on caspase-3 protein [F(4, 45) = 48.50, *p* = 0.0001], Bax protein [F(4, 45) = 34.60, *p* = 0.0001], and Bcl-2 protein [F(4, 45) = 21.00, *p* = 0.0001]..

Figure 6 illustrates the effects of ACR and/or ATRA treatments on caspase-3 (A), Bax (B), and Bcl-2 (C) levels in testicular tissues. ACR treatment significantly increased caspase-3 levels compared with the control (CTR) group (8.380 ± 0.783 vs. 1.010 ± 0.208 ng/mg), representing an increase of approximately 729.7% (*p* = 0.0001), and Bax levels (273.9 ± 24.14 vs. 83.51 ± 3.724 ng/mg), reflecting an increase of approximately 227.9% (*p* = 0.0001). In contrast, Bcl-2 levels were significantly reduced in the ACR group compared with the CTR group (88.91 ± 9.409 vs. 227.3 ± 15.17 ng/mg), corresponding to a decrease of approximately 60.8% (*p* = 0.0001).

Conversely, ATRA pre-treatment prior to ACR exposure significantly reduced caspase-3 levels compared with the ACR group (3.560 ± 0.3724 vs. 8.380 ± 0.783 ng/mg), indicating a reduction of approximately 57.7% (*p* = 0.0001), and Bax levels (106.3 ± 6.447 vs. 273.9 ± 24.14 ng/mg), corresponding to a decrease of approximately 61.2% (*p* = 0.0001). In addition, ATRA pre-treatment significantly increased Bcl-2 levels compared with the ACR group (186.5 ± 6.205 vs. 88.91 ± 9.409 ng/mg), representing an increase of approximately 109.7% (*p* = 0.001).

Similarly, mRNA expression levels of apoptosis markers caspase-3, Bax and Bcl-2 were shown in **supplementary Fig. 1A**,** B and C**. ACR intoxication induced a striking upregulation of testicular Caspase-3 mRNA expression (5.29 ± 0.50) relative to control rats (1.01 ± 0.21), corresponding to a 424.0% increase (*p* < 0.05), indicative of robust activation of the executioner apoptotic pathway. ATRA monotherapy maintained Caspase-3 expression at levels slightly below control values (0.79 ± 0.09), confirming its intrinsic anti-apoptotic potential under physiological conditions. Co-administration of ATRA with ACR significantly suppressed ACR-induced Caspase-3 overexpression by 43.5% (2.99 ± 0.64 vs. 5.29 ± 0.50; *p* < 0.05), reflecting meaningful attenuation of apoptotic signaling. Additionally, testicular Bax mRNA expression was significantly elevated in ACR-intoxicated rats (1.82 ± 0.25) compared to controls (1.00 ± 0.04), reflecting an 82.0% increase consistent with ACR-induced pro-apoptotic signaling (*p* < 0.0001). ATRA monotherapy slightly reduced Bax expression below baseline levels (0.79 ± 0.07; −21.0% vs. CTR), demonstrating its intrinsic capacity to suppress apoptotic gene transcription under non-toxic conditions. Co-administration of ATRA with ACR significantly attenuated Bax overexpression by 29.7% relative to the ACR group (1.28 ± 0.08 vs. 1.82 ± 0.25; *p* < 0.0001).

In contrast to the pro-apoptotic pattern observed for Bax, testicular Bcl-2 mRNA expression was markedly downregulated in ACR-intoxicated rats (0.489 ± 0.076) relative to controls (1.000 ± 0.118), representing a 51.1% reduction consistent with ACR-mediated suppression of pro-survival signaling (*p* < 0.001). ATRA monotherapy significantly upregulated Bcl-2 expression (1.411 ± 0.092; +41.1% vs. CTR; *p* < 0.0001), affirming its capacity to reinforce the anti-apoptotic transcriptional program. Co-administration of ATRA with ACR substantially restored Bcl-2 levels (0.808 ± 0.068), yielding a 65.2% increase over the ACR group (*p* < 0.0001).

### Histopathological examination of the testicular tissue specimens

**Fig. 7:**
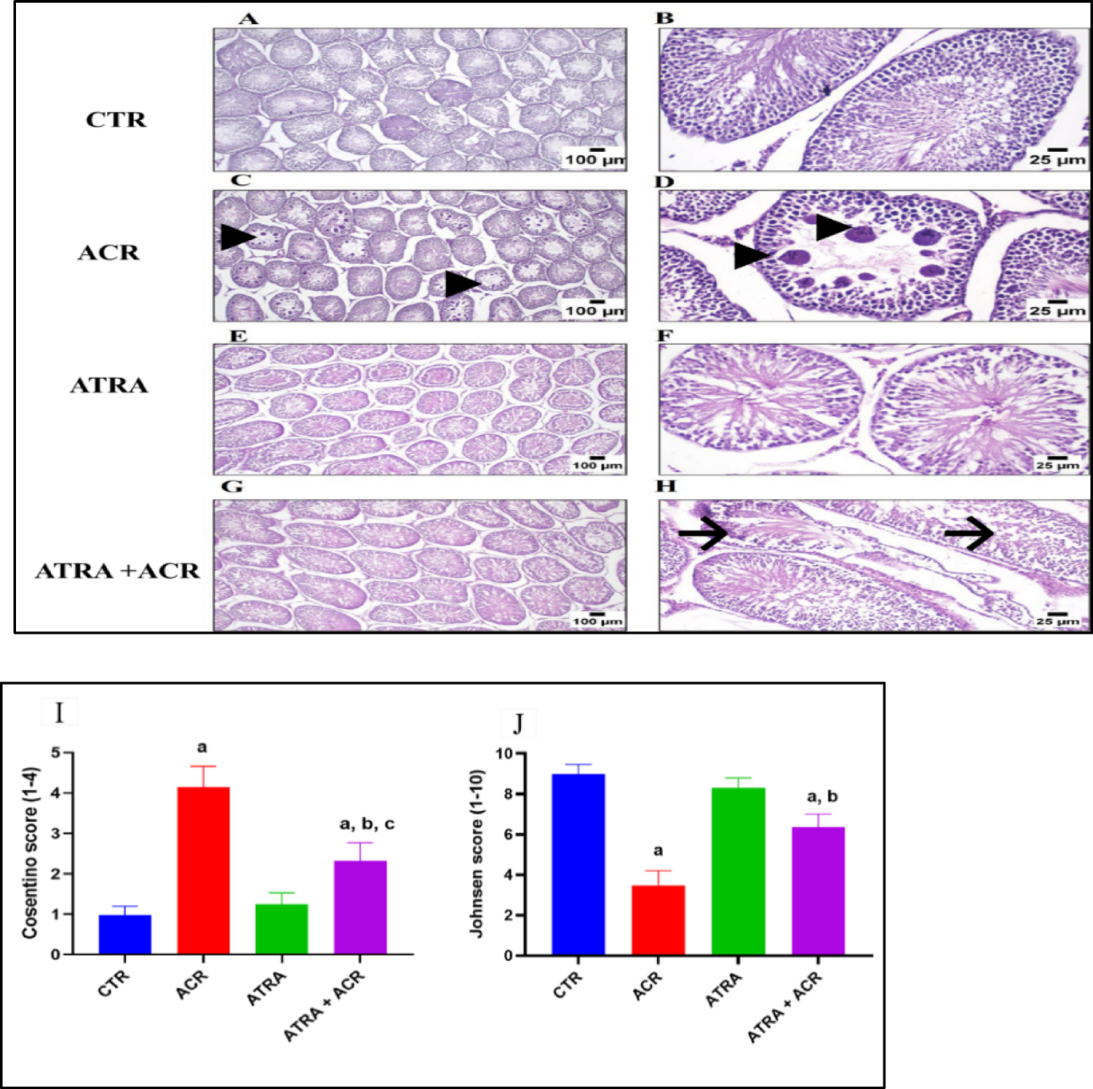
Effect of ATRA and/or ACR on the histopathological features of the testes. (A and B) Normal structure of the testis is seen in the control group. (C and D) The appearance of multinucleated giant cells in the seminiferous tubules and atrophied seminiferous tubules in the ACR group (Arrow heads). (E and F). Normal structure is seen in the ATRA group. (G and H) Testis structure in the ATRA + ACR shows improvement of testicular tissue toward normal appearance, except fewer number of seminiferous tubules displaying mild vacuolar degeneration of the affected tubules (arrow) (H&E staining, scale bar = 100 μm at left and 25 μm at right). (I) Johnsen’s and (J) Cosentino score of groups are presented as mean ± SD. One-way ANOVA identified significant group differences in Johnsen score [F(3, 36) = 22.3, *p* = 0.001] and Cosentino grade [F(3, 36) = 30.5, *p* = 0.001].

The control group **(**Fig. 7A **and B)** showed no detectable alterations, with normal histological architecture of mature seminiferous tubules with complete spermatogenesis, along with intact Sertoli cells and interstitial Leydig cells. In contrast, the ACR-treated group **(**Fig. 7C **and D)** demonstrated marked pathological changes, including numerous multinucleated giant cell formations within the seminiferous tubules, tubular atrophy, and severely congested blood vessels. The ATRA-treated group **(**Fig. 7E **and F)** showed no histopathological alterations, maintaining normal, intact seminiferous tubules with a complete spermatogenic series. Meanwhile, the ACR+ATRA-treated group **(**Fig. 7G **and H)** exhibited largely preserved histological structures in most seminiferous tubules, although a few tubules displayed mild vacuolar degeneration.

To confirm the above histological findings, scoring was done by Johnsen’s and Cosentino scoring system **(**Fig. 7I **and J)**. Histopathological evaluation of spermatogenesis using Johnsen’s scoring system revealed a profound reduction in mean scores in ACR-intoxicated rats (3.48 ± 0.72) compared to controls (8.98 ± 0.48), reflecting a 61.2% decline indicative of severe disruption of the spermatogenic process (*p* < 0.001). ATRA-treated rats maintained near-normal Johnsen scores (8.28 ± 0.52), with no statistically significant difference from the CTR group, confirming the absence of intrinsic retinoid toxicity on testicular histoarchitecture. Importantly, co-administration of ATRA with ACR resulted in a substantial recovery of Johnsen scores (6.37 ± 0.64), representing an 83.0% improvement relative to the ACR group (*p* < 0.05), though scores remained moderately below control values, suggesting partial rather than complete histological restoration.

ACR intoxication caused a marked elevation in Cosentino scoring (4.15 ± 0.51) compared to control rats (0.98 ± 0.21), representing a 322.8% increase (*p* < 0.001). ATRA monotherapy maintained scoring close to control values (1.25 ± 0.28), with no statistically significant difference from the CTR group. Notably, co-administration of ATRA with ACR significantly attenuated ACR-induced elevation, reducing Cosentino scoring by 44.1% relative to the ACR group (2.32 ± 0.46 vs. 4.15 ± 0.51; *p* < 0.001).

## Discussion

The study assessed the protective effects of All-trans retinoic acid (ATRA) against ACR-induced testicular toxicity in male rats. ACR exposure significantly impaired reproductive function, reducing body weight, testicular weight, sperm count, motility, LDH-X and serum testosterone, FSH and LH levels. It also induced oxidative stress, inflammation, and apoptosis, shown by increased MDA levels and decreased antioxidant enzyme activities (GSH, SOD, CAT). Elevated pro-inflammatory cytokines (TNF-α, IL-1β, IL-6) and pro-apoptotic markers (caspase-3, Bax) correlated with histopathological testicular damage, including seminiferous tubule degeneration.

Co-administration of ATRA improved ACR-induced adverse effects by restoring antioxidant enzyme activities and reducing lipid peroxidation, relieving oxidative stress. It decreased inflammatory cytokines, favoring cell survival by lowering caspase-3 and Bax while increasing Bcl-2 levels. ATRA also enhanced testicular histology, preserving seminiferous tubule structure and reducing histopathological abnormalities, thus mitigating ACR-induced reproductive toxicity. The findings align with prior research on the protective effects of ATRA against toxic agents such as cisplatin. For instance, a study on cisplatin-induced testicular damage demonstrated that ATRA improved sperm parameters, reduced oxidative stress markers (total oxidant status and oxidative stress index), and ameliorated histopathological changes through its antioxidant properties^25^. Notably, both studies highlight the ability of ATRA to restore spermatogenesis and reduce oxidative damage. However, while cisplatin predominantly targets germ cells, leading to spermatogenesis arrest^25^, acrylamide induces broader damage involving inflammation and apoptosis pathways. This distinction underscores the versatility of ATRA as a protective agent across different toxicological models.

The LDH-X isoenzyme is the most important enzyme which has regulatory roles in mechanisms of metabolic processes that are required for spermatogenesis and sperm viability and motility^37^^38^. In parallel to our previous study^16^, the present findings showed that the administration of ACR notably reduced LDH-X activity, indicating testicular and sperm damage and this observation reinforces the rationale for the decrease in sperm count and motility. On the other hand, pretreatment with ATRA preserved LDH-X activity and sperm count and motility suggesting that ATRA has a specific role in improving the spermatogenesis. Therefore, the restoration of healthy LDH-X levels serves as a compelling biochemical indicator that the ATRA intervention successfully protected the later stages of spermatogenesis from the toxic insult such as ACR.

The retinoic acid receptor alpha (RARα) is essential for the testes’ regular operation, especially for controlling spermatogenesis. Sertoli cells (SCs) and germ cells are among the cell types in the rat testis that have been found to express RARα, indicating a complicated interaction between spermatogenesis and testicular function^39^^40^. In the present study, ACR exposure resulted in marked downregulation of RAR-α expression in testicular tissue, as evidenced by immunofluorescence analysis. The suppression of RAR-α likely contributes to disrupted spermatogenesis, degeneration of seminiferous tubules, and decreased testosterone synthesis observed in the ACR-treated rats. The inhibition of RAR-α signaling may impair the transcription of RA-responsive genes, including Stra8 (stimulated by retinoic acid gene 8), which is essential for the transition of spermatogonia to pre-leptotene spermatocytes^41^. Disruption of RAR-α signaling has previously been shown to cause asynchrony of the spermatogenic cycle and progressive germ cell depletion^34^, supporting the mechanistic connection between RAR-α suppression and ACR-induced testicular damage.

It was proposed that both testosterone production by Leydig cells and proliferation of Sertoli cells are controlled by RAR-α during the fetal period^42^. Additionally, blocking RA signaling in Leydig cells resulted in increased permeability of the blood-testis barrier, decreased levels of the steroidogenic enzyme cytochrome P450 17A1 and decreased testosterone levels^43^. In our study, the restoration of testosterone levels by ATRA in the ACR+ATRA group suggests that RAR-α signaling may contribute to preserving Leydig cell function under toxic stress. The decline in testosterone induced by ACR exposure may be attributed to Leydig cell weakening, triggered by elevated LH secretion^44^. The observed reduction in serum FSH levels in the ACR-exposed group is consistent with prior reports documenting that ACR acts as an endocrine disruptor, suppressing the hypothalamic-pituitary-gonadal axis and impairing the secretion of gonadotropins, including FSH^44^^45^. The partial restoration of FSH levels in the ACR+ATRA group indicates ATRA’s cytoprotective effect on Sertoli cells. FSH and retinoic acid (RA) signaling interact in the seminiferous epithelium, influencing spermatogonial differentiation and meiotic entry^46^. Retinoid signaling via RAR-α is crucial for germ cell differentiation, with ATRA promoting Sertoli cell differentiation and modulating proliferation signals^47^.

The current study attributes the modulatory effects to ATRA’s multifaceted actions, particularly its antioxidant properties that counteract oxidative stress from ACR. ATRA restores GSH, SOD, and CAT activities, reducing MDA levels, thereby enhancing the antioxidant defense system and minimizing free radical-induced lipid peroxidation and cellular injury. Additionally, ATRA shows strong anti-inflammatory effects, as evidenced by the significant reduction in TNF-α, IL-1β, and IL-6 levels, which helps preserve testicular architecture and function. Moreover, ATRA demonstrated notable anti-apoptotic properties in the current model. The balance between pro-apoptotic proteins (caspase-3 and Bax) and the anti-apoptotic protein Bcl-2 is critical for determining cell fate in testicular tissues. ATRA administration reduced the expression of pro-apoptotic markers while upregulating Bcl-2, thereby promoting cell survival and reducing germ cell loss. The interplay between RAR-α signaling and apoptosis provides further mechanistic insight into the protective role of ATRA. Previous studies demonstrated that activation of RARs suppresses pro-apoptotic gene expression and enhances the expression of anti-apoptotic genes such as Bcl-2^48^. In the present study, ATRA decreased caspase-3 and Bax levels while restoring Bcl-2, consistent with anti-apoptotic actions mediated via RAR-α activation.

Several other investigations have shown that ATRA has anti-inflammatory effects across various models, indicating its protective role beyond reproductive toxicity. In murine ulcerative colitis, ATRA modulated the TLR4/NF-κB pathway, decreasing pro-inflammatory mediators like TNF-α and NOS2, thus reducing colonic inflammation^49^. ​ Similarly, in a rat model of acute lung injury, ATRA enhanced macrophage phagocytic activity and lowered IL-6 and IL-1β levels by inhibiting the CD14/TLR4 pathway^50^. Additionally, in vitro, ATRA also suppressed TNF-α and nitric oxide production in activated macrophages^51^.

Another line of evidence is derived from gut models in which ATRA was found to inhibit virus-induced inflammation in epithelial cells through the suppression of RLRs/NF-κB signaling cascade^52^. and to repair the intestinal barrier and decrease inflammation in adenoma-related diarrhea^53^. In addition, systemic effects of ATRA have been shown in mice that ATRA scavenged reactive oxygen and nitrogen species, decreased lipid peroxidation, acute and chronic inflammation and majorly inhibited tumor correlated angiogenesis^54^. These findings collectively highlight the broad anti-inflammatory and antioxidant potential of ATRA in various pathological contexts.

In contrast to our findings, excessive intake of ATRA can adversely impact animal reproduction. High doses of retinoic acid can harm animal reproduction, causing testicular degeneration and impaired spermatogenesis^55^, with effects on fetal testicular development, germ cell reduction, and disrupted meiotic regulation^56^. The apparent discrepancy can be explained by the differences in dose, timing, and biological circumstances. The toxicity data support the notion that ATRA can be helpful when administered effectively and harmful only when mis-dosed, rather than contradicting our findings. They further emphasize the significance of regulated RA homeostasis.

In line with our earlier study, the histological analyses, which showed a marked disruption of the normal testicular architecture and the formation of multinucleated giant cells in the seminiferous tubules and atrophied seminiferous tubules, further validate ACR-induced testicular toxicity. When ATRA and ACR were administered together, multinucleated giant cells disappeared and the testicular structures returned to normal, much like in the control group. These results are in accordance with the study of Yucel et al., 2019^25^. This highlights the cytoprotective role of ATRA against ACR-induced testicular toxicity.

## Limitations and future directions

The study acknowledges a limitation due to the 14-day duration of ACR and ATRA administration, while rat spermatogenesis requires about 48–52 days for full evaluation. Future research should focus on long-term effects of ACR and ATRA to understand chronic toxicity and protective mechanisms, including sperm viability and morphology analyses for a comprehensive evaluation. The reliance on RAR-α expression data necessitates further molecular analyses to explore gene expression and signaling pathways related to retinoids. Dose-response studies are vital for identifying optimal ATRA concentrations, and assessing effects on specific testicular cell types will provide deeper insights, along with translational studies for human relevance.

## Conclusion

**Fig. 8:**
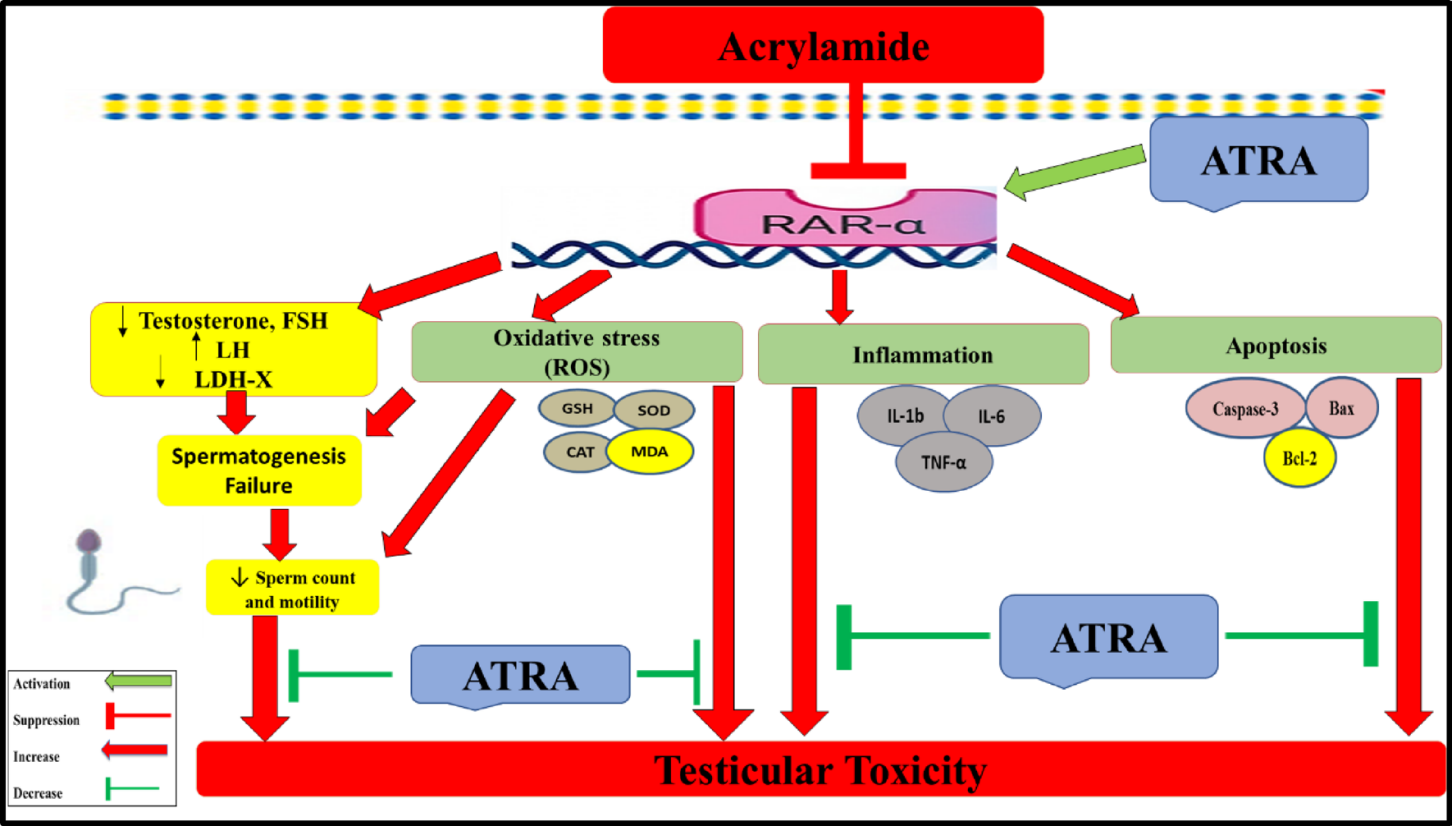
A representative diagram depicting the potential mechanisms by which ATRA modulates the harmful effects of acrylamide on testicular tissue.

Collectively, our findings suggest that ATRA attenuates ACR-induced testicular toxicity and is associated with restoration of RAR-α expression, alongside improvements in antioxidant defenses (SOD, GSH and CAT), inflammatory markers (TNF-α, IL-6 and IL-1β), and apoptotic parameters (Caspase-3, Bax and Bcl-2). The observed ability of ATRA to enhance antioxidant defenses, suppress inflammation, and reduce apoptotic markers, alongside the associated upregulation of RAR-α expression, highlights its potential as a candidate for preserving male fertility in populations at risk of acrylamide exposure as illustrated in Fig. 8. Based on these results, we advocate for additional mechanistic research employing functional receptor studies, gene expression analyses, pathway-specific analyses and longer experimental durations to further clarify the role of ATRA and RAR-α signaling in male reproductive health.

## Electronic Supplementary Material

Below is the link to the electronic supplementary material.

Supplementary Material 1

## Data Availability

The data used for this study are available from the corresponding author upon request.

## References

[1] Braun, O. et al. Spotlight on the life cycle of acrylamide-based polymers supporting reductions in environmental footprint: review and recent advances. *Molecules* **27**, 42. 10.3390/molecules27010042 (2021). 35011281 10.3390/molecules27010042 PMC8746853

[2] Al-Kindi, S., Al-Bahry, S., Al-Wahaibi, Y., Taura, U. & Joshi, S. Partially hydrolyzed polyacrylamide: enhanced oil recovery applications, oil-field produced water pollution, and possible solutions. *Environ. Monit. Assess.* **194**, 875. 10.1007/s10661-022-10569-9 (2022). 36227428 10.1007/s10661-022-10569-9 PMC9558033

[3] Tepe, Y. & Çebi, A. Acrylamide in environmental water: a review on sources, exposure, and public health risks. *Exposure Health*. **11**, 3–12. 10.1007/s12403-017-0261-y (2019).

[4] Adimas, M. A., Abera, B. D., Adimas, Z. T., Woldemariam, H. W. & Delele, M. A. Traditional food processing and Acrylamide formation: A review. *Heliyon* **10**, e30258. 10.1016/j.heliyon.2024.e30258 (2024). 38720707 10.1016/j.heliyon.2024.e30258 PMC11076960

[5] Bušová, M., Bencko, V., Laktičová, K. V., Holcátová, I. & Vargová, M. Risk of exposure to acrylamide. *Cent. Eur. J. Public Health*. **28**, S43–S46. 10.21101/cejph.a6177 (2020). 33069180 10.21101/cejph.a6177

[6] Liu, Y. et al. Chronic acrylamide exposure resulted in dopaminergic neuron loss, neuroinflammation and motor impairment in rats. *Toxicol. Appl. Pharmcol.* **451**, 116190. 10.1016/j.taap.2022.116190 (2022). 10.1016/j.taap.2022.116190 35917840

[7] Pennisi, M. et al. Neurotoxicity of acrylamide in exposed workers. *Int. J. Environ. Res. Public Health*. **10**, 3843–3854. 10.3390/ijerph10093843 (2013). 23985770 10.3390/ijerph10093843 PMC3799507

[8] Pourentezari, M. Acrylamide and Male Infertility: Exploring the Implication for Reproductive Health. *J. Infertility Reproductive Biology*. **12**, 1–9 (2024).

[9] Çebi, A. *in Acrylamide in food* 65–93 (Elsevier, 2024). 10.1016/C2021-0-01613-8

[10] Ahmed, M. M. et al. Reproductive Injury in Male Rats from Acrylamide Toxicity and Potential Protection by Earthworm Methanolic Extract. *Anim. (Basel)*. 12. 10.3390/ani12131723 (2022). 10.3390/ani12131723 PMC9264786 35804622

[11] Authority, E. F. S., Benford, D., Bignami, M. & Chipman, J. K. Ramos Bordajandi, L. Assessment of the genotoxicity of acrylamide. *EFSA J.* **20**, e07293. 10.2903/j.efsa.2022.7293 (2022). 35540797 10.2903/j.efsa.2022.7293 PMC9069548

[12] Seify, M. et al. Impacts of Acrylamide on testis and spermatozoa. *Mol. Biol. Rep.* **51**, 739. 10.1007/s11033-024-09677-1 (2024). 38874886 10.1007/s11033-024-09677-1

[13] Kandemir, F. M. et al. Protective effects of morin against acrylamide-induced hepatotoxicity and nephrotoxicity: A multi-biomarker approach. *Food Chem. Toxicol.* **138**, 111190. 10.1016/j.fct.2020.111190 (2020). 32068001 10.1016/j.fct.2020.111190

[14] Zhao, M. et al. The involvement of oxidative stress, neuronal lesions, neurotransmission impairment, and neuroinflammation in acrylamide-induced neurotoxicity in C57/BL6 mice. *Environ. Sci. Pollut. Res.* **29**, 41151–41167. 10.1007/s11356-021-18146-2 (2022). 10.1007/s11356-021-18146-2 35088269

[15] Singh, J., Kumar, D., Rachamalla, M. & Jangra, A. Toxic Effects of Acrylamide and Their Underlying Mechanisms. *Sustainable Dev. Goals Towards Environ. Toxic. Green. Chem.* 225–248. 10.1007/978-3-031-77327-3_12 (2025).

[16] Mokhlis, H. A. et al. Hydrogen sulfide alleviates acrylamide-induced testicular toxicity in male rats. *Toxicol. Environ. Health Sci.* **15**, 41–51. 10.1007/s13530-022-00156-3 (2023).

[17] Rajeh, N. A. Systematic Review of Acrylamide-Induced Toxicity and Oxidative Stress in Humans and Animals: Antioxidant Strategies for Male Reproductive Dysfunction. *Int. J. Agric. Biosci.* **15**, 646–668. 10.47278/journal.ijab/2025.216 (2026).

[18] Carazo, A. et al. Vitamin A update: forms, sources, kinetics, detection, function, deficiency, therapeutic use and toxicity. *Nutrients* **13**, 1703. 10.3390/nu13051703 (2021). 34069881 10.3390/nu13051703 PMC8157347

[19] Pareek, A. et al. Retinoic acid in Parkinson’s disease: Molecular insights, therapeutic advances, and future prospects. *Life Sci.*10.1016/j.lfs.2024.123010 (2024). 123010. 39181315 10.1016/j.lfs.2024.123010

[20] Deng, Q. & Chen, J. Potential therapeutic effect of all-trans retinoic acid on atherosclerosis. *Biomolecules* **12**, 869. 10.3390/biom12070869 (2022). 35883425 10.3390/biom12070869 PMC9312697

[21] Borges, G. S. M., Lima, F. A., Carneiro, G., Goulart, G. A. C. & Ferreira, L. A. M. All-trans retinoic acid in anticancer therapy: how nanotechnology can enhance its efficacy and resolve its drawbacks. *Expert Opin. Drug Deliv.* **18**, 1335–1354. 10.1080/17425247.2021.1919619 (2021). 33896323 10.1080/17425247.2021.1919619

[22] Hunsu, V. O., Facey, C. O., Fields, J. Z. & Boman, B. M. Retinoids as chemo-preventive and molecular-targeted anti-cancer therapies. *Int. J. Mol. Sci.* **22**, 7731. 10.3390/ijms22147731 (2021). 34299349 10.3390/ijms22147731 PMC8304138

[23] Ewees, M. G., Abdelghany, T. M., Abdel-Aziz, A. A. & Abdel-Bakky, M. S. All-trans retinoic acid mitigates methotrexate-induced liver injury in rats; relevance of retinoic acid signaling pathway. *Naunyn Schmiedebergs Arch. Pharmacol.* **388**, 931–938. 10.1007/s00210-015-1130-5 (2015). 25971792 10.1007/s00210-015-1130-5

[24] Elsayed, A. M., Abdelghany, T. M., Akool el, S., Abdel-Aziz, A. A. & Abdel-Bakky, M. S. All-trans retinoic acid potentiates cisplatin-induced kidney injury in rats: impact of retinoic acid signaling pathway. *Naunyn Schmiedebergs Arch. Pharmacol.* **389**, 327–337. 10.1007/s00210-015-1193-3 (2016). 26659823 10.1007/s00210-015-1193-3

[25] Yucel, C. et al. Protective Effect of All-Trans Retinoic Acid in Cisplatin-Induced Testicular Damage in Rats. *World J. Mens Health*. **37**, 249–256. 10.5534/wjmh.180105 (2019). 30799561 10.5534/wjmh.180105 PMC6479087

[26] Yokoi, K., Uthus, E. O. & Nielsen, F. H. Nickel deficiency diminishes sperm quantity and movement in rats. *Biol. Trace Elem. Res.* **93**, 141–154. 10.1385/BTER:93:1-3 (2003). 12835498 10.1385/BTER:93:1-3:141

[27] Tan, S. et al. Zearalenone-induced aberration in the composition of the gut microbiome and function impacts the ovary reserve. *Chemosphere* **244**, 125493. 10.1016/j.chemosphere.2019.125493 (2020). 32050327 10.1016/j.chemosphere.2019.125493

[28] Uchiyama, M. & Mihara, M. Determination of malonaldehyde precursor in tissues by thiobarbituric acid test. *Anal. Biochem.* **86**, 271–278. 10.1016/0003 (1978). -2697(78)90342-1. 655387 10.1016/0003-2697(78)90342-1

[29] Beutler, E. & Yeh, M. K. Erythrocyte glutathione reductase. *Blood* **21**, 573–585. 10.1182/blood.V21.5.573.573 (1963). 13967896

[30] Aebi, H. in *Oxygen Radicals in Biological Systems* Vol. 105 *Methods in Enzymology* 121–126Academic Press, (1984). 6727658

[31] Nishikimi, M., Appaji, N. & Yagi, K. The occurrence of superoxide anion in the reaction of reduced phenazine methosulfate and molecular oxygen. *Biochem. Biophys. Res. Commun.* **46**, 849–854. 10.1016/S0006-291X(72)80218-3 (1972). 4400444 10.1016/s0006-291x(72)80218-3

[32] Abdel-Bakky, M. S., Hammad, M. A., Walker, L. A. & Ashfaq, M. K. Silencing of tissue factor by antisense deoxyoligonucleotide prevents monocrotaline/LPS renal injury in mice. *Arch. Toxicol.* **85**, 1245–1256. 10.1007/s00204-011-0663-8 (2011). 21327618 10.1007/s00204-011-0663-8

[33] Bancroft, J. D. & Gamble, M. *Theory and practice of histological techniques* (Elsevier health sciences, 2008).

[34] Johnsen, S. G. Testicular biopsy score count–a method for registration of spermatogenesis in human testes: normal values and results in 335 hypogonadal males. *Hormone Res. Paediatrics*. **1**, 2–25. 10.1159/000178170 (1970). 10.1159/000178170 5527187

[35] Cosentino, M. J., Nishida, M., Rabinowitz, R. & Cockett, A. T. Histological changes occurring in the contralateral testes of prepubertal rats subjected to various durations of unilateral spermatic cord torsion. *J. Urol.* **133**, 906. 10.1016/S0022-5347(17)49278-0 (1985). 3989935 10.1016/s0022-5347(17)49278-0

[36] ŞEKERCİ, Ç. et al. Protective effects of ellagic acid on testicular ischemia-reperfusion injury in rats. *J. Urol. Surg.* **10**10.4274/jus.galenos.2022.2022.0049 (2023).

[37] Odet, F. et al. Lactate dehydrogenase C and energy metabolism in mouse sperm. *Biol. Reprod.* **85**, 556–564. 10.1095/biolreprod.111.091546 (2011). 21565994 10.1095/biolreprod.111.091546 PMC3159538

[38] Saeed, B., Baban, R. & Al-Nasiri, U. Lactate dehydrogenase C4 (LDH-C4) is essential for the sperm count and motility: A case-control study. *Baghdad J. Biochem. Appl. Biol. Sci.* **2**, 146–159. 10.47419/bjbabs.v2i03.65 (2021).

[39] Chung, S. S., Sung, W., Wang, X. & Wolgemuth, D. J. Retinoic acid receptor alpha is required for synchronization of spermatogenic cycles and its absence results in progressive breakdown of the spermatogenic process. *Dev. Dyn.* **230**, 754–766. 10.1002/dvdy.20083 (2004). 15254909 10.1002/dvdy.20083 PMC3785309

[40] Li, X. et al. The roles of retinoic acid in the differentiation of spermatogonia and spermatogenic disorders. *Clin. Chim. Acta*. **497**, 54–60. 10.1016/j.cca.2019.07.013 (2019). 31302099 10.1016/j.cca.2019.07.013

[41] Endo, T., Freinkman, E., de Rooij, D. G. & Page, D. C. Periodic production of retinoic acid by meiotic and somatic cells coordinates four transitions in mouse spermatogenesis. *Proc. Natl. Acad. Sci.* **114**, E10132–E10141. 10.1073/pnas.1710837114 (2017). 29109271 10.1073/pnas.1710837114 PMC5703301

[42] Condrea, D. et al. Retinoic acid receptor alpha is essential in postnatal Sertoli cells but not in germ cells. *Cells* **11**, 891. 10.3390/cells11050891 (2022). 35269513 10.3390/cells11050891 PMC8909012

[43] Luo, J., Pasceri, P., Conlon, R. A., Rossant, J. & Giguere, V. Mice lacking all isoforms of retinoic acid receptor beta develop normally and are susceptible to the teratogenic effects of retinoic acid. *Mech. Dev.* **53**, 61–71. 10.1016/0925-4773(95)00424-6 (1995). 8555112 10.1016/0925-4773(95)00424-6

[44] Yildirim, S. et al. Selenium reduces acrylamide-induced testicular toxicity in rats by regulating HSD17B1, StAR, and CYP17A1 expression, oxidative stress, inflammation, apoptosis, autophagy, and DNA damage. *Environ. Toxicol.* **39**, 1402–1414. 10.1002/tox.23996 (2024). 37987225 10.1002/tox.23996

[45] Sengul, E., Gelen, V., Yildirim, S., Cinar, İ. & Aksu, E. H. Effects of naringin on oxidative stress, inflammation, some reproductive parameters, and apoptosis in acrylamide-induced testis toxicity in rat. *Environ. Toxicol.* **38**, 798808. 10.1002/tox.23728 (2023). 10.1002/tox.23728 36598108

[46] Khanehzad, M., Abbaszadeh, R., Holakuyee, M., Modarressi, M. H. & Nourashrafeddin, S. M. FSH regulates RA signaling to commit spermatogonia into differentiation pathway and meiosis. *Reproductive Biology Endocrinol.* **19**10.1186/s12958-020-00686-w (2021). 4. 10.1186/s12958-020-00686-w PMC7789255 33407539

[47] Nicholls, P. K., Harrison, C. A., Rainczuk, K. E., Vogl, A. W. & Stanton, P. G. Retinoic acid promotes Sertoli cell differentiation and antagonises activin-induced proliferation. *Mol. Cell. Endocrinol.* **377**, 33–43. 10.1016/j.mce.2013.06.034 (2013). 23831638 10.1016/j.mce.2013.06.034

[48] Balmer, J. E. & Blomhoff, R. Gene expression regulation by retinoic acid. *J. Lipid Res.* **43**, 1773–1808. 10.1194/jlr.R100015-JLR200 (2002). 12401878 10.1194/jlr.r100015-jlr200

[49] Rafa, H. et al. All-trans retinoic acid modulates TLR4/NF‐κB signaling pathway targeting TNF‐α and nitric oxide synthase 2 expression in colonic mucosa during ulcerative colitis and colitis associated cancer. *Mediators of inflammation* 7353252, (2017). 10.1155/2017/7353252 (2017). 10.1155/2017/7353252 PMC5376956 28408791

[50] Li, S., Lei, Y., Lei, J. & Li, H. All-trans retinoic acid promotes macrophage phagocytosis and decreases inflammation via inhibiting CD14/TLR4 in acute lung injury. *Mol. Med. Rep.* **24**, 868. 10.3892/mmr.2021.12508 (2021). 34676874 10.3892/mmr.2021.12508 PMC8554390

[51] Mehta, K., McQueen, T., Tucker, S., Pandita, R. & Aggarwal, B. B. Inhibition by all-trans-retinoic acid of tumor necrosis factor and nitric oxide production by peritoneal macrophages. *J. Leucocyte Biology*. **55**, 336–342. 10.1002/jlb.55.3.336 (1994). 10.1002/jlb.55.3.336 8120450

[52] Pu, J. et al. All-trans retinoic acid attenuates transmissible gastroenteritis virus-induced inflammation in IPEC-J2 Cells via suppressing the RLRs/NF-κB signaling pathway. *Front. Immunol.* **13**, 734171. 10.3389/fimmu.2022.734171 (2022). 35173714 10.3389/fimmu.2022.734171 PMC8841732

[53] Priyamvada, S. et al. All-trans retinoic acid counteracts diarrhea and inhibition of downregulated in adenoma expression in gut inflammation. *Inflamm. Bowel Dis.* **26**, 534–545. 10.1093/ibd/izz249 (2020). 31634391 10.1093/ibd/izz249 PMC7456978

[54] Siddikuzzaman & Grace, V. M. B. Antioxidant potential of all-trans retinoic acid (ATRA) and enhanced activity of liposome encapsulated ATRA against inflammation and tumor-directed angiogenesis. *Immunopharmacol. Immunotoxicol.* **35**, 164–173. 10.3109/08923973.2012.736520 (2013). 23116338 10.3109/08923973.2012.736520

[55] Comitato, R. et al. Impairment of spermatogenesis and enhancement of testicular germ cell apoptosis induced by exogenous all-trans-retinoic acid in adult lizard Podarcis sicula. *J. Exp. Zool. Comp. Exp. Biol.* **305**, 288–298. 10.1002/jez.a.264 (2006). 10.1002/jez.a.264 16432891

[56] Jorgensen, A. et al. Ex vivo culture of human fetal gonads: manipulation of meiosis signalling by retinoic acid treatment disrupts testis development. *Hum. Reprod.* **30**, 2351–2363. 10.1093/humrep/dev194 (2015). 26251460 10.1093/humrep/dev194 PMC5513102

