# Supplementary material for: ATRA-mediated RAR-α activation attenuates acrylamide-induced testicular toxicity
Source: Sci Rep. 2026 May 7;16:14644. doi: 10.1038/s41598-026-50168-z (PMC13153413; doi:10.1038/s41598-026-50168-z)
Supplement: Supplementary file 1 — Supplementary Material 1 [file 41598_2026_50168_MOESM1_ESM.docx]

| **Gene Name** | **Primer** | **Length (bp)** |
| --- | --- | --- |
| **Caspase-3** | **Forward: ACTGGAATGTCAGCTCGCAA**  **Reverse: GCAGTAGTCGCCTCTGAAGA** | **270** |
| **Bax** | **Forward: TTTCATCCAGGATCGAGCAG**  **Reverse: AATCATCCTCTGCAGCTCCA** | **154** |
| **Bcl-2** | **Forward: GACTTTGCAGAGATGTCCAG**  **Reverse: TCAGGTACTCAGTCATCCAC** | **214** |
| **β-Actin** | **Forward: CAGCCTTCCTTCTTGGGTATG**  **Reverse: AGCTCAGTAACAGTCCGCCT** | **360** |

**Supplementary Table 3: Nucleotide sequences and product size of primers used in reverse transcription-polymerase chain reaction.**

**
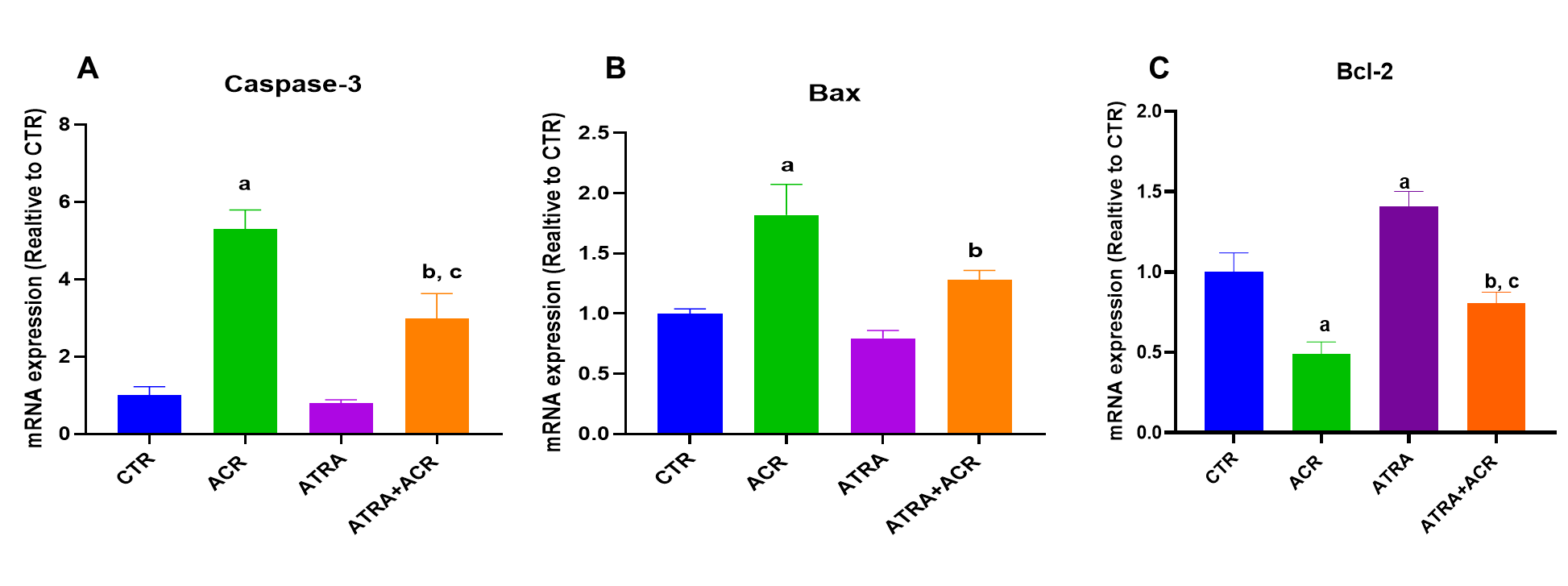
**

**Supplementary Figure 1.** **Effects of ATRA and/or ACR on mRNA** **expression level of apoptosis biomarkers:**

mRNA expression level of Caspases-3 (A), Bax (B), and BCL-2 (C) levels in testicular tissues of rats treated with acrylamide (ACR). Results expressed as the mean ± SD (n = 10). ^a^ Significant from CTR. ^b^ Significant from ACR. ^c^ Significant from ATRA. at P ≤ 0.05. Significant at 5% for ANOVA. One-way ANOVA revealed significant treatment effects on caspase-3 protein [F(3, 36) = 18.30, p = 0.0001], Bax protein [F(3, 36) = .00, p = 0.0001], and Bcl-2 protein [F(3, 36) = 21.00, p = 0.0001].

Also, sperm viability and morphology should be performed in the future work
